# Pharmacokinetics of Octreotide in Patients With Cirrhosis and Portal Hypertension; Relationship Between the Plasma Levels of the Analogue and the Magnitude and Duration of the Reduction in Corrected Wedged Hepatic Venous Pressure

**DOI:** 10.1155/1998/17436

**Published:** 1998-08

**Authors:** S. A. Jenkins, D. M. Nott, J. N. Baxter

**Affiliations:** 1 University Department of Surgery Royal Liverpool University Hospital Liverpool L7 8XP United Kingdom; 2 Department of Surgery Postgraduate Medical School Morriston Hospital Swansea Morriston SA6 6NL USA; 3 Academic Department of Surgery Chelsea and Westminster Hospital London SW 10 9NH United Kingdom

## Abstract

In healthy subjects octreotide is largely metabolised by the liver suggesting that the plasma half-life of the somatostatin analogue may be prolonged in patients with hepatic dysfunction. The aim of this study was therefore (a) to determine the pharmacokinetics of octreotide following its subcutaneous injection in 6 patients with cirrhosis and portal hypertension and (b) compare the magnitude and duration of the effects of intravenous administration of 250 μg somatostatin and 50 μg octreotide on corrected wedged hepatic venous pressure (WHVP) and to relate the findings to the plasma levels of the analogue 1h after administration in 13 patients with cirrhosis and portal hypertension. Following subcutaneous administration of 50 μg octreotide the circulating half life (range 2.4 to 4.79 h) was prolonged whereas the clearance (range 2.101 to 4.775 L/h) was decreased compared to healthy controls. Intravenous bolus administration of 25 μg somatostatin or 50 μg octreotide resulted in a reduction in WHVP of approximately the same magnitude and duration despite appreciable quantities of the analogue in the blood lh after administration (1944 ± 226 pg/ml). These results indicate that the circulating half-life of octreotide is prolonged in cirrhotics suggesting that the dosage regimens should be modified in such patients to avoid accumulation of the analogue in the blood which may result in undesirable side-effects or toxicity. Furthermore, since the magnitude and duration of the reduction in WHVP elicited by IV octreotide is similar to that obseved with somatostatin, the analogue, like the native hormone, must be administered by continuous IV infusion to produce a sustained response and hence a therapeutic effect in the management of acute variceal bleeding.


*HPB Surgery*, 1998, Vol. 11, pp. 13–21
Reprints available directly from the publisher
Photocopying permitted by license only

© 1998 OPA (Overseas Publishers Association) N.V.
Published by license under
the Harwood Academic Publishers imprint,
part of The Gordon and Breach Publishing Group.
Printed in India.

### Pharmacokinetics of Octreotide in Patients with Cirrhosis and Portal Hypertension; Relationship Between the Plasma Levels of the Analogue and the Magnitude and Duration of the Reduction in Corrected Wedged Hepatic Venous Pressure

S. A. JENKINS*,**, D. M. NOTT† and J. N. BAXTER*

*University Department of Surgery, Royal Liverpool University Hospital, Liverpool L7 8XP*

*(Received in final form 3 June 1997)*

**In healthy subjects octreotide is largely metabolised by the liver suggesting that the plasma half-life of the somatostatin analogue may be prolonged in patients with hepatic dysfunction. The aim of this study was therefore (a) to determine the pharmacokinetics of octreotide following its subcutaneous injection in 6 patients with cirrhosis and portal hypertension and (b) compare the magnitude and duration of the effects of intravenous administration of 250 μg somatostatin and 50 μg octreotide on corrected wedged hepatic venous pressure (WHVP) and to relate the findings to the plasma levels of the analogue 1 h after administration in 13 patients with cirrhosis and portal hypertension. Following subcutaneous administration of 50 μg octreotide the circulating half life (range 2.4 to 4.79 h) was prolonged whereas the clearance (range 2.101 to 4.775 L/h) was decreased compared to healthy controls. Intravenous bolus administration of 250 μg somatostatin or 50 μg octreotide resulted in a reduction in WHVP of approximately the same magnitude and duration despite appreciable quantities of the analogue in the blood 1 h after administration (1944 ± 226 pg/ml). These results indicate that the circulating half-life of octreotide is prolonged in cirrhotics suggesting that the dosage regimens should be modified in such patients to avoid accumulation of the analogue in the blood which may result in undesirable side-effects or toxicity. Furthermore, since the magnitude and duration of the reduction in WHVP elicited by IV octreotide is similar to that obseved with somatostatin, the analogue, like the native hormone, must be administered by continuous IV infusion to produce a sustained response and hence a therapeutic effect in the management of acute variceal bleeding.**

*Keywords:* Pharmacokinetics, octreotide, somatostatin, cirrhosis, wedged hepatic venous pressure

### INTRODUCTION

Somatostatin, a naturally occuring 14-amino acid peptide has a plethora of effects on gastrointestinal function leading to the suggestion

* Present address: Department of Surgery, Postgraduate Medical School, Morriston Hospital, Morriston Swansea SA6 6NL.
† Present address: Academic Department of Surgery, Chelsea and Westminster Hospital, London SW 10 9NH.
**Corresponding author.

that it might have a therapeutic role in a variety of gastrointestinal indications. However, since somatostatin has a short half-life (1–2 mins) [1] and requires intravenous administration to maintain therapeutic levels, longer acting analogues have been developed. Octreotide, one such analogue, is an octapeptide in which the 4 amino acid sequence essential for the biological activity of the native hormone is retained. However, incorporation of *N*-terminal phenyalanine, *C*-terminal amino alcohol, *D*-tryptophan and a cysteine bridge makes the molecule very resistant to metabolic degration [2, 3] and consequently, octreotide has a much longer circulating half-life than somatostatin in healthy volunteers [4, 5]. In normal healthy volunteers octreotide is largely metabolised by the liver, the hepatic excretion being in the order of 30–40% [5]. No data is available on the plasma half-life of octreotide in cirrhotic patients but it is reasonable to assume that the hepatic extraction is reduced according to the degree of liver dysfunction and that the circulating half-life is likely to be prolonged compared to normal subjects. Consequently, the relationship between dosage regimens and the clinical effects of octreotide are likely to be different in cirrhotic patients compared to healthy volunteers. Since interest is accumulating in the use of octreotide for the control of the acute variceal hemorrhage and in the long-term management of portal hypertension, we have investigated the circulatory half-life of octreotide following its subcutaneous administration in cirrhotics. Furthermore, we also studied the relationship between the magnitude and duration of response of octreotide on corrected wedged hepatic venous pressure (WHVP) and the circulating levels of the analogue after intravenous administration.

### MATERIALS AND METHODS

This study was approved by the Royal Liverpool University Hospital Ethical Committee and all patients gave written informed consent.

### Subcutaneous Administration of Octreotide

Six patients (4 males and 2 females), receiving 50 μg octreotide s.c. twice daily to prevent recurrent varieceal hemorrhage were studied 4 weeks after commencing treatment with the analogue. These patients were participating in a randomised controlled trial and had already undergone a baseline measurement of corrected wedged hepatic venous pressure (WHVP) prior to being commenced on octreotide (50 μg.sc.bd). Furthermore, since all patients were scheduled to have their WHVP measured at the end of study (six months), it was considered unethical to subject them to a further haemodynamic investigation four weeks into the trial. Since octreotide administered subcutaneously acts as a slow release preparation, there is no information on the temporal relationships between the time of administration and the reductions in corrected WHVP in patients already receiving the analogue. Consequently, it would not have been practical to measure corrected WHVP for the duration of this part of the study (12 h). One patient had severe (Child's C), 2 moderate (Child's B) and 3 mild (Child's A) liver dysfunction. Following an overnight fast, blood samples were removed immediately prior to a subcutaneous injection of 50 μg octreotide and then at 2 hourly intervals for the following 12 h. The blood samples were collected in heparinised bottles containing Trasylol, centrifuged at 0°C, the plasma snap frozen and stored at −20°C for subsequent analysis of the octreotide levels.

### INTRAVENOUS ADMINISTRATION OF OCTREOTIDE

Thirteen patient with cirrhosis and portal hypertension admitted for a thorough investigation of the etiology and severity of their liver disease underwent measurements of their corrected wedged hepatic venous pressure (WHVP).

Two introducer sheaths (Cordis 8F, Cordis Corporation, Miami, USA) were inserted into the right femoral vein under local anaesthesia. A 7F occlusion ballon Sidewinder catheter (Cordis Corporation, Miami, USA) was guided into a hepatic vein under fluoroscopic control. With the ballon inflated, confirmation of wedging was obtained by the absence of the reflux into the inferior vena cava following the injection of 2 ml of intravenous contrast. A straight 7F Cordis catheter was inserted into the femoral vein via the second introducer and guided under fluoroscopic control until its tip lay free in the inferior vena cava (IVC) at the level of the ballon catheter for the measurement of free hepatic vein pressure. The right femoral artery was cannulated with a 7F Cordis cannula via a 8F Cordis introducer. Pressure were recorded with isolated Medex 860 transducers (Medex Medical Inc. Rossendale, UK) connected via amplifiers to an Electromed multichannel chart recorder (Electromed UK Ltd., Letchworth, Herts, UK). The midchest region was chosen as the zero level for transducer calibration. Corrected wedged hepatic venous pressure was calculated by subtracting the free pressure (IVC catheter) from the wedged (balloon catheter). Using two catheters allowed continuous measurement of wedged and free hepatic venous pressures during administration of the vasoactive drugs or saline. When not being used to measure wedged hepatic venous pressure the balloon of the Sidewinder catheter was deflated and the catheter withdrawn 1–2 cm. The free pressure measurements obtained from both catheters were compared to ensure that the inferior cava pressure measurements were representative of the free wedged hepatic venous pressure. The Sidewinder catheter was then reintroduced into the hepatic vein, the balloon inflated and confirmation of wedging reconfirmed as described above.

Heart rate was recorded using a pressure sensitive cuff connected to a Cardiocaps monitor (Dataex Ltd, Helsinki, Finland).

A small catheter was introduced into an antecubital vein for the administration of the vasoactive drugs or saline. Ideally, the best experimental design would have been to randomise the administrations of somatostatin and octreotide in a double-blind manner. However, at the time of study we expected octreotide to have a prolonged duration of action on hepatic haemodynamics and therefore we considered it was not practical to administer the drugs in a double-blind randomised fashion. Consequently, all patients received bolus injections of saline, 250 µg somatostatin, saline, 50 µg octreotide and saline consecutively, all in a volume of 2 ml. All injections were given 10 min after the haemodynamic parameters had stabilised following the previous administration of saline or vasoactive drug and during this period the balloon on the Sidewinder catheter was deflated. The patients were unaware of whether or not they were receiving saline, somatostatin or octreotide. The magnitude and duration of the effects of somatostatin, octreotide and saline on wedged and free hepatic venous pressures, arterial blood pressure and heart rate were recorded.

One hour after completion of the investigations a blood sample was removed from all patients and stored for subsequent determination of octreotide levels.

### Analytical Methods

The concentration of octreotide in the plasma samples was measured using a specific radioimmunoasay (Sandoz Pharmaceuticals, Basle, Switzerland) as previously described [5]. The sensitivity of the assay was 5 pg/ml, the cross reactivity with somatostatin less than 0.01% and the intra- and inter-assay coefficients less than 10%. The plasma half-life of octreotide was calculated by linear regression over the period 2–12 h after administration. The area under the curve (AUC) 2–12 h after administration of octreotide was calculated by the Trapezoidal rule correcting for the declining basal levels by subtracting Co/K and for the AUC 2–12 h by subtracting $C_{12}$/K where:-

Co and $C_{12}$=concentrations at times 0 and 12 h

$$K = \frac{0.693}{t^{1/2}} \frac{0.693}{T^{1/2}}$$

These allowances used to calculate the AUC virtually cancel each other indicating a steady state. Clearance (CL) was derived from Dose/AUC 2–12 h and the volume of distribution (Vd) from CL/K.

### Statistical Analysis

Differences in corrected WHVP levels before and after administration of somatostatin, octreotide and saline were evaluated using a Mann Whitney test for paired data.

### RESULTS

### Subcutaneous Administration of Octreotide

All patients had been receiving 50 µg octreotide s.c. b.d. for 4 weeks with the last injection being administered 12 h prior to commencement of the study. Consequently, all patients had detectable baseline levels of octreotide in the plasma immediately prior to the s.c. injection of 50 µg of the analogue for the pharmacokinetic study. Furthermore, the baseline octreotide levels were highest in the patient with severe hepatic dysfunction and lowest in the 3 with good liver function (Tab. I).

Peak plasma concentrations of octreotide were observed 2 h after subcutaneous administration of 50 µg octreotide and thereafter declined in all patients. The lowest peak levels of octreotide were observed in patients with good liver function and the highest in the one patient with severe hepatic dysfunction (Tab. I). The plasma half-life of octreotide in the 6 patients was variable (range 2.4 to 4.89 h) being appreciably shorter in patients with good liver function than those with moderate or severe hepatic dysfunction (Tab. II). Nevertheless, overall, the plasma half-life of octreotide in the six patients (3.44 ± 1.01 h; mean ± SD) was increased and the clearance decreased (3.04 ± 0.9 L/h mean ± SD) compared to those previously reported for normal healthy subjects [5].

TABLE I Baseline and peak plasma levels of octreotide in the 6 patients participating in the pharmacokinetic study

| Patient | Child's grade | Plasma octreotide levels (pg/ml) Basal | Peak |
|---|---|---|---|
| 1 | A | 148 | 2588 |
| 2 | A | 88 | 2198 |
| 3 | A | 178 | 2092 |
| 4 | B | 396 | 2942 |
| 5 | B | 720 | 3276 |
| 6 | C | 1195 | 3511 |

### Intravenous Administration of Octreotide and Somatostatin

Approximately 15–20 seconds after intravenous bolus administration of either 250 µg somatostatin or 50 µg octreotide, wedged and corrected wedged hepatic venous pressure began to decrease, the maximum reductions being observed approximately 50–60 seconds after administration of both drugs (Tab. III). Thereafter, both wedged and corrected wedged

TABLE II Pharmacokinetic variables of octreotide (50 µg s.c.) in six patients with cirrhosis and portal hypertension

| Patient | Child's grade | t1/2 (h) | K ($h^{-1}$) | AUC (ng/ml.h) | Clearance (L/h) | Volume of distribution (L) |
|---|---|---|---|---|---|---|
| 1 | A | 2.40 | 0.271 | 10.47 | 4.775 | 17.62 |
| 2 | A | 2.76 | 0.251 | 17.21 | 2.892 | 11.51 |
| 3 | A | 2.56 | 0.288 | 16.55 | 3.021 | 10.47 |
| 4 | B | 4.03 | 0.172 | 18.08 | 2.765 | 16.07 |
| 5 | B | 4.04 | 0.172 | 18.49 | 2.704 | 15.77 |
| 6 | C | 4.89 | 0.142 | 23.79 | 2.101 | 14.82 |

TABLE III The effects and duration of action of somatostatin, octreotide and saline on hepatic venous pressure, mean arterial blood pressure and pulse in 13 patients with cirrhosis and portal hypertension

| | Saline | | Somatostatin | | Saline | | Octreotide | | Saline | |
|---|---|---|---|---|---|---|---|---|---|---|
| | Before | After | Before | After | Before | After | Before | After | Before | After |
| Wedged hepatic venous pressure (mm Hg) | 31.6+1.9 | 32.1+1.9 | 31.6+1.8 | 27.2+1.7** | 31.5+2.0 | 31.7+2.0 | 31.8+1.8 | 29+2.1* | 31.6+1.8 | 31.8+1.9 |
| Free hepatic venous pressure (mm Hg) | 8.4+1.8 | 8.7+1.9 | 8.3+1.6 | 8.6+1.8 | 8.4+1.6 | 8.4+1.7 | 8.4+1.9 | 8.6+1.7 | 8.5+1.8 | 8.6+1.9 |
| Corrected hepatic venous pressure (mm Hg) | 23.1+1.5 | 23.4+1.7 | 23.3+1.8 | 18.6+1.8** | 23.1+1.9 | 23.3+1.8 | 23.4+1.7 | 18.4+1.5** | 23.1+1.6 | 23.2+1.5 |
| Mean arterial blood pressure (mm Hg) | 103+5 | 104+4 | 105+4 | 121+8* | 103+4 | 102+5 | 101+4 | 115+5* | 102+5* | 103+4 |
| Pulse beats (min) | 75+3 | 76+4 | 78+3 | 65+3* | 75+4 | 75+4 | 76+3 | 63+3* | 77+3 | 76+4 |
| Duration of hepatic venous response (min) | – | | 1.9+0.4 | | – | | 2.3+0.6 | | – | |

Results are expressed as mean ± SEM.
$^*p < 0.01$.
$^{**}p < 0.001$.
The changes shown after administration of somatostatin, octreotide and saline are the maximal responses from the baseline for each patient.

hepatic venous pressures began to increase. Thus, somewhat surprisingly there was no significant difference in the duration of the reduction of wedged or corrected wedged hepatic venous pressures following somatoatin or octreotide administration (Tab. III), despite the presence of appreciable amounts of octreotide in the plasma (1944 ± 226 pg/ml) one hour after administration. Saline administered before, between or following the administration of somatostatin and octreotide had no significant effect on wedged or corrected wedged hepatic venous pressures.

Between administrations of somatostatin, octreotide and saline when the balloon was deflated and the Sidewinder catheter withdrawn from the wedged position, there was excellent agreement between the free pressures recorded from this catheter and the other lying free in the inferior cava, the differences in the readings not exceeding 0.2 mm Hg. Somatostatin, octreotide and saline had no significant effect on the free hepatic venous pressure.

Approximately 30–40 seconds after intravenous bolus administration of either 250 µg somatostain or 50 µg octreotide mean arterial pressure began to increase, the maximal response occurring 70–80 seconds after administration of both drugs (Tab. III). Thereafter, arterial pressure began to decrease. There was no significant difference in the duration of the increase in mean arterial blood pressure following somatostatin (2.1 ± 0.6 min) or octreotide (2.3 ± 0.5 min) administration. Saline had no effect on arterial blood pressure (Tab. III).

Forty to fifty seconds after intravenous administration of 250 µg somatostatin or 50 µg octreotide, the heart rate began to decrease, the maximum reduction occurring approximately 80–90 seconds after administration of both drugs (Tab. III). Thereafter, the pulse rate began to return to normal. There was no significant difference in the duration of the bradycardia following somatostatin (2.0 ± 0.6 min) or octreotide (2.3 ± 0.5 min) administration. Saline had no significant effect on the pulse rate (Tab. III). No major side effects of somatostatin or octreotide were observed in this study.

### DISCUSSION

The results of this study demonstrate that following subcutaneous administration the plasma half-life of octreotide is increased and the clearance decreased in cirrhotic patients compared to those previously reported for normal healthy controls [4, 5] using the same radioimmunoassay kit. Furthermore, there appeared to be a considerable variation in the plasma half-life of octreotide in cirrhotic patients according to the severity of the liver disease. Thus in cirrhotics with severe (Child's C) or moderate (Child's B) hepatic dysfunction the plasma half-life of octreotide was appreciably longer than in patients with mild disease (Child's A), although the small number of patients studied limits any definitive conclusions. These observations are perhaps not unexpected since the liver is the principal site for the elimination of octreotide, the hepatic excretion being estimated to be between 30–40% in normal healthy volunteers [5]. Consequently it is reasonable to assume that the hepatic excretion of octreotide varies according to the severity of the liver disease, a hypothesis supported by the findings of the present study. Further support for this suggestion is provided by the finding that half of the dosage of octreotide resulted in almost identical arterial concentrations of the analogue in cirrhotic patients compared to the levels observed in normal healthy controls after a full dose [6]. Further studies are, however, required to fully establish the relationship between the degree of hepatic dysfunction and the plasma half-life of octreotide.

The six patients who participated in the pharmacokinetic part of this study had been receiving 50 μg of octreotide s.c. b.d. for 4 weeks prior to investigation. In all these patients appreciable levels of octreotide were present in the baseline blood samples, i.e., 12 h after the last injection of the analogue prior to the study. Furthermore, the highest plasma levels were observed in those with moderate (Child's B) or severe (Child's C) liver disease. These observations suggest that even with a relatively low dosage regime, i.e., 50 μg octreotide s.c. b.d. there is accumulation of the analogue in the blood, particularly in patients with moderate or severe liver disease, presumably as a result of the increase in the plasma half-life. These observations have obvious implications for the there apeutic use of octreotide in cirrhotic patients. Thus, particularly during prolonged periods of administration in cirrhotic patients, dosage regimens should be modified to avoid accumulation of octreotide in the blood which may result in undersirable side-effects or toxicity.

Intravenous bolus administrations of either 250 μg somatostatin or 50 μg octreotide resulted in a significant decrease in wedged and corrected wedged hepatic venous pressures. These results are therefore in accord with previous observations indicating that intravenous administration of both somatostatin and octreotide and subcutaneous administration of the analogue decrease portal pressure [7–14]. However, it should be pointed out that other reports suggests that octreotide and somatostatin have no effect on corrected WHVP in portal hypertensive patients [15–17]. These conflicting observations on the effects of somatostatin and octreotide on corrected WHVP are difficult to reconcile but may be related to the amount given, the mode of administration and the severity of the portal hypertension. Nevertheless, the consensus of opinion is that both somatostatin and octreotide reduce corrected WHVP in cirrhotic patients by eliciting a splanchnic vasoconstriction thereby reducing portal flow and portal pressure [18]. The increase in arterial blood pressure and decrease in heart rate elicited by both octreotide and somatostatin in the present study occurred after the wedged and corrected wedged hepatic venous pressures began to decrease suggesting that they may be secondary reflex responses to compensate for the increase in splanchnic vasoconstriction.

The precise mechanism whereby somatostatin and octreotide elicit splanchnic vasoconstriction is not known. The rapidity of onset of the reduction in corrected WHVP following somatostatin or octreotide administration supports the suggestion that both compounds act directly on receptors on the smooth muscle of the splanchnic vasculature [19]. Alternatively, somatostatin and octreotide may elicit vasoconstriction by inhibiting the release and end-organ action of vasoactive substances responsible for maintaining the hyperdynamic circulation in portal hypertensive patients. Clearly, further work is required to establish more precisely the mechanism of action of somatostatin and octreotide on hepatic and splanchnic haemodynamics. Nevertheless, irrespective of their mode of action, the reduction in portal venous inflow, portal pressure and azygos blood flow secondary to splanchnic vasoconstriction is presumed to be mechanism whereby both drugs are effective in controlling the acute variceal bleed.

In the present study a bolus injection of 250 μg somatostatin or 50 μg octreotide elicited a reduction in corrected WHVP of approximately the same magnitude. Furthermore, the duration of the response to octreotide was not significantly greater than that elicited by somatostatin, despite the presence of appreciable amounts of the analogue in the blood 1 h after administration. The short duration of action of octreotide on WHVP was perhaps surprising since other effects of the analogue such as the suppression of growth hormone or growth hormone releasing factor are prolonged and proportional to the blood levels [20]. However, it is now evident that there are a family of receptors that bind somastostatin and octreotide with different binding affinities [21, 22, 23]. Furthermore, the somatostatin receptor sub-types have variable but specific structural requirements to facilitate binding with either somatostatin or its analogues [21, 22, 23]. Differences in the somatostatin receptor sub-type responsible for a specific action of somatostatin would offer an explanation for differences between the pharmacodynamic characteristics of octreotide with respect to its effect on growth hormone and insulin release [24]. Thus, after a single dose of octreotide in rhesus monkeys, the inhibition of growth hormone release persists for much longer periods than that of insulin [24]. The somatostatin receptors in gastrointestinal tissue appear to consist of at least two subtypes viz. high affinity receptors similar to those found in the cerebral cortex and low affinity, high capacity subtypes to which analogues such as octreotide bind poorly or not at all [23]. If these low affinity somatostatin receptors are involved in the events leading to a reduction in corrected WHVP by somatostatin or octreotide, the low binding of the analogue to such sites would offer an explanation for the relatively short duration of response. However, this hypothesis requires confirmation by identification of the somatostatin receptor subtypes involved in reducing WHVP. Nevertheless, regardless of the mechanisms involved, the results of this study clearly suggest that in terms of reducing portal pressure, octreotide is not long-acting and requires intravenous administration to produce a sustained decrease in portal pressure. This suggestion is supported by the results of clinical trials on the efficacy of octreotide in controlling acute variceal bleeding. Thus, one such trial reported that intravenous administration of octreotide (25 μg/ h) was more efficacious than glypressin plus nitroglycerin (86% and 62% respectively) in controlling bleeding over a 12 h period [25]. Over the next 36 h of the trial during which octreotide was given subcutaneously, more patients in the analogue-treated group experienced rebleeding than in the glypressin-nytroglycerin group so that the overall control of bleeding was not significantly different between the 2 groups over the 48 h assessment period (55% and 48% overall control of bleeding respectively) [25]. In contrast, two trials have reported that intravenous octreotide is as good as one course of injection sclerotherapy in controlling variceal bleeding and preventing recurrence over a 48 h trial period [26– 27].

In summary, the results of this study indicate that the plasma half-life of octreotide in cirrhotic patients is prolonged, presumably as a result of diminished hepatic excretion. However, in terms of reducing portal pressure, the duration of action of octreotide is not significantly longer than that of somatostatin. Consequently, like somatostatin, octreotide has to be administered intravenously to produce a sustained response and hence a therapeutic effect in the management of the acute variceal bleed.

### Acknowledgments

We are grateful to Sandoz Pharmaceuticals Ltd. Camberley, UK for providing the octreotide and its specific radioimmunoassay kit and to Serono Pharmaceuticals, UK for the supply of somatostatin. We would also like to thank Mr. T. Hine, Department of Clinical Chemistry, Royal Liverpool Hospital for carrying out the radioimmunoassay.

### References

[1] Sheppard, M., Shapiro, B., Berelowitz, M. and Pimstone, B. (1979). Metabolic clearance and plasma half disappearance time of exogenous somatostatin in man. *J. Clinic Endocrinol. and Metab.*, **48**, 50–53.

[2] Veber, D. F., Freidinger, R. M., Schwenk-Perlow, R. *et al.* (1981). A potent cyclic hexapeptide analogue of somatostatin. *Nature*, **292**, 55–58.

[3] Rosenthal, L. E., Yamashiro, D. J., Rivier, J. *et al.* (1983). Structure-activity relationship of somatoastatin analogues in the rabbit ileum and rat colon. *J. Clin. Invest.*, **71**, 840–849.

[4] del Pozo, E., Neufield, M., Schlutr, K. *et al.* (1986). Endocrine profile of a long acting somatostatin derivative SMS 201–995. Study in normal volunteers following subcutaneous administration. *Acta Endocrinologica*, **111**, 433–439.

[5] Kutz, K., Nuesch, E. and Rosenthaler, J. (1986). Pharmacokinetics of SMS 201–995 in healthy subjects. *Scan J. Gastroenterology*, **21** (sup 119), 65–72.

[6] Erikson, L. S., Brundin, T., Soderlund, C. and Wahren, J. (1987). Haemodynamic effects of a long-standing somatostatin analogue in patients with liver cirrhosis. *Scand. J. Gastroent.*, **22**, 919–925.

[7] Tyden, G., Samnegaard, H., Thulin, L. and Friman, L. (1978). Treatment of bleeding oesophageal varices with somatostatin. *New Engl. J. Med.*, **22**, 919–925.

[8] Bories, P., Pomier-Layragues, G., Chotard, J. P., Jacob, C. and Michel, H. (1980). Lasomotostatine dimue l'hypertension portale chez le cirrhotique. *Gastroenterologie et Biologique*, **4**, 616–617.

[9] Bosch, J., Kravetz, D. and Rhodes, J. (1981). Effects of somatostatin on hepatic and systemic haemodynamics in patients with cirrhosis of the liver; comparison with vasopressin. *Gastroenterology*, **80**, 518–525.

[10] Naeije, R., Hallemans, R., Mois, P., Melot, C. H. and Reding, P. (1982). Effects of vasopressin and somatostatin on haemodynamics and blood gases in patients with liver cirrhosis. *Crit. Care Med.*, **10**, 578–582.

[11] Eriksson, L. S., Law, D., Sato, Y. and Wahren, J. (1984). Influence of somatostatin on splanchnic haemodynamics in patients with liver cirrhosis. *Clinic Physiol.*, **4**, 5–11.

[12] Navasa, M., Bosch, J., Chesta, J. *et al.* (1988). Haemodynamics effect of subcutaneous administration of SMS 201–995, a long-acting somatostatin analogue in patients with cirrhosis and portal hypertension. *J. Hepatol.*, **64**, 123.

[13] Pringle, S. D., McKee, R. F., Garden, O. J., Lorimer, P. R. and Carter, D. C. (1988). The effects of a long-acting somatostatin analogue on portal and systemic hameodynamics in cirrhosis. *Alimentary Pharmacol and Therapeutics*, **2**, 451–459.

[14] Merkel, C., Gatta, A., Zuin, R. *et al.* (1985). Effect of somatostatin on splanchnic haemodynamics in patients with cirrhosis of the liver and portal hypertension. *Digestion*, **32**, 92–98.

[15] Sonnenberg, G. E., Keller, U., Perruchoud, A., Burkhardt, D. and Gyr, K. (1981). Effect of somatostatin on splanchnic haemodynamics in patients with cirrhosis of the liver and in normal subjects. *Gastroenterology*, **80**, 526–532.

[16] Burroughs, A. K., Santana, J. A., Dux, T., Dick, R. and McIntyre, N. (1985). Effect of a long-acting analogue of somatostatin (SMS 201–995) on hepatic venous pressures and azygos blood flow in cirrhotics. *Gastroenterology*, **26**, 562.

[17] McCormick, P. A., Dick, R., Siringo, S. *et al.* (1990). Octreotide reduces azygos blood flow in cirrhotic patients with portal hypertension. *Eur. J. Gastroenterol and Hepatol.*, **2**, 489–492.

[18] Hanisch, E., Doertenbach, J. and Usadel, K. H. (1992). Somatostatin in acute bleeding oesophageal varices. *Pharmacology and Rationale for use. Drugs*, **44** (sup 2), 24–35.

[19] Samnegaard, H., Thulin, L., Andreen, M. *et al.* (1979). Circulatory effects of somatostatin on anaesthetized dogs. *Acta Chir Scand*, **145**, 209–212.

[20] Marbach, P., Briner, U., Lemaire, M., Schweitzer, A. and Teraski, T. (1993). From somatostatin to sandostatin; pharmacodynamics and pharmacokinetics. *Digestion*, **5** (sup 1), 9–13.

[21] Yamada, Y., Post, S. R., Wang, K., Tager, H. S., Bell, G. I. and Seino, S. (1992). Cloning and functional characterisation of a family of human and mouse somatostatin receptors expressed in brain; gastrointestinal tract and liver. *Proc. Natl. Acad. Sci.*, USA, **78**(87), 3930–3934.

[22] Srikant, C. B. and Patel, Y. C. (1992). Somatostatin receptors. Identification and characterisation in brain membranes. *Proct. Natl. Acad. Sci.*, USA, **78**, 1183–1193.
[23] Miller, C. V., Preston, Sr., Woodhouse, L. F., Farmery, S. M. and Primrose, J. H. (1993). Somatostatin binding in human gastrointestinal tissues: effects of cations and somatostatin analogues. *Gut.*, **34**, 1351–1356.
[24] Marbach, P., Andrew, H., Azria, M. *et al.* (1988). Chemical structure and pharmacodynamic properties of SMS 201–995 (Sandostatin) in the treatment of acromegaly. In "The Treatment of Acromegaly", Berlin, Springer, 53–60.
[25] Silvain, C., Carpentier, S., Sautereau, S. *et al.* (1991). A randomized trial of glypressin plus transdermal nitroglycerin versus octreotide in the control of acute variceal haemorrhage. *Hepatology*, **14**, 1339.
[26] Jenkins, S. A., Copeland, G., Sutton, R., Kingsnorth, A. N. and Shields, R. (1992). Octreotide (SMS) versus sclerotherapy for variceal bleeding: preliminary results. *Br. J. Surg.*, **79**, 1224.
[27] Sung, J. J. Y., Chung, S., Lai, C.-W. *et al.* Octreditide infusion or emegency aclecthocopy for variocal hasmarhage. *Lancet*, **342**, 637–641.

